# NLRP3 inflammasome promoted the malignant progression of prostate cancer via the activation of caspase-1

**DOI:** 10.1038/s41420-021-00766-9

**Published:** 2021-12-20

**Authors:** Zheng Xu, Hao Wang, Zhiqiang Qin, Feng Zhao, Liuhua Zhou, Luwei Xu, Ruipeng Jia

**Affiliations:** Department of Urology, Nanjing First Hospital, Nanjing Medical University, Nanjing, Jiangsu 210006 China

**Keywords:** Cancer microenvironment, Urological cancer

## Abstract

It is widely accepted that inflammation is an important risk for the development of prostate cancer (PCa). The objective of this study was designed to investigate the potential molecular mechanism of NLR family, pyrin domain-containing protein 3 (NLRP3) inflammasome in the malignant progression of PCa. The expression level of NLRP3 was evaluated by quantitative real-time polymerase chain reaction (qRT-PCR) and fluorescence in situ hybridization. The effects of NLRP3 in the development of PCa by applying gain- and loss-of-function assays in LNCaP and PC3 cell lines were detected by CCK-8, TUNEL, and Transwell migration assays. The underlying mechanism of NLRP3 and caspase-1 in PCa was examined by the rescue experiments, western blotting, and qRT-PCR assays. In addition, the promoting effect of NLRP3 inflammasome was performed with an animal subcutaneous tumorigenesis experiment in vivo. The upregulation of NLRP3 was confirmed in PCa tissues and cell lines. Functionally, using CCK-8, TUNEL, and Transwell migration assays, these results showed that activation of NLRP3/caspase-1 inflammasome by LPS + ATP could enhance the ability of proliferation and migration; and decrease the apoptosis of LNCaP and PC3 cell lines. Western blotting assay showed that the activation of caspase-1 would increase after the stimulation of NLRP3 inflammasome by LPS + ATP. Moreover, the overexpression of NLRP3 promoted, while the knockdown of NLRP3 inhibited the malignant progression in PCa cell lines by positively regulating caspase-1. In addition, the rescue experiments revealed the association among NLRP3 and caspase-1, which showed that the overexpression vectors/inhibitors of caspase-1 could reverse the effect of knockdown/overexpression of NLRP3 in PCa cell lines in vitro. Finally, In in vivo experiment, the suppression of NLRP3 knockdown impaired tumor growth of PCa. Collectively, these results indicated that NLRP3 inflammasome played a vital role in promoting the malignant progression of PCa via the activation of caspase-1. Together, our findings provided insight into the mechanisms of NLRP3/caspase-1 inflammasome and revealed an alternative and potential target for the clinical diagnosis and treatment of PCa.

## Introduction

Prostate cancer (PCa) is one of the most common malignancies among men worldwide, with an estimated 191,930 new cases and 33,330 new deaths of PCa in the United States in 2020 [1, 2]. The incidence of PCa cases has been steadily increasing in the last decade, and varies among different ethnic backgrounds, which is relatively low incidence in the developing countries, compared with a few Western developed countries [3, 4]. Despite the recent progression of PCa, it is a significant medical problem for the men affected, which is a combination of genetic factors, lifestyle, nutrition, environmental factors, and so on [5, 6]. However, with the over-treatment of inherently benign disease and inadequate therapies for metastatic PCa, the diagnosis and treatment technology of PCa still need to be further improved [7, 8]. Therefore, the early diagnosis and intervention of PCa would help to choose the best treatment options of PCa, including radical prostatectomy, hormone therapy, chemotherapy, and radiotherapy [8]. A growing body of evidence highlights the importance of chronic inflammation for malignant proliferation, migration, angiogenesis, and resistance to chemotherapy in a variety of cancers [9]. There is therefore an urgent need to explore cancer-related inflammation to find new treatments that are more effective and have fewer side effects.

It has been reported that inflammation involved in the progression of tumorigenesis and carcinogenesis [9–11]. On the one hand, acute inflammation could protect the body against infectious pathogens. On the other hand, chronic inflammation has been found that it could play a vital role in tissue impairment, DNA damage, epigenetic changes, and genetic, might lead to the initiation and progression of cancer [10, 12]. Inflammasomes are intracellular multi-protein complexes that initiate tissue inflammatory responses to various danger signals [13, 14]. Pyrin domain-containing protein 3 (NLRP3) of the NLR family is the most well-defined and widely studied inflammasome. It is activated by a two-step model called “priming” and “activation”. Once activated by NF-κB activation signal, it results in the expression of restriction proteins (such as NLRP3 receptors) activated by endogenous NLRP3 inflammasome or microbial [15]. NLRP3 inflammasome is activated in a second step by pore-forming toxins, viral RNA, damage-associated molecular patterns, such as ATP or particular matter [16]. NLRP3 act as platforms for the activation of caspase-1 and maturation of inflammatory cytokines including IL-1β and IL-18 after being activated [17]. Caspase-1-related cell death is known as pyroptosis and involves inflammation and tissue repair; IL-18 and Il-1 β also play important roles in inflammatory response [17, 18].

Previous studies have shown that the importance of NLRP3 inflammasome in the initiation and progression of cancer has been well documented [19, 20]. Chung et al. suggested that upregulated mitochondrial oxidative phosphorylation components are strongly associated with NLRP3 inflammasome activation in nasopharyngeal carcinoma [20]. However, molecular mechanism and the biological function of NLRP3 inflammasome in PCa need to be further studied. Therefore, the identification of NLRP3 regulated target genes and their signaling pathways is the focus of research on tumor-associated inflammation. In this study, we found that the expression level of NLRP3 was upregulated in PCa tissues and cell lines. In addition, the downregulation of NLRP3 inflammasome impaired the proliferation, apoptosis, and migration of PCa cell lines. Furthermore, NLRP3 inflammasome epigenetically activated the expression level of caspase-1, thus promoting the malignant progression of PCa. Together, illuminating the roles and mechanisms of NLRP3 inflammasome would provide novel insights for the diagnosis and treatment of PCa.

## Results

### High expression of NLRP3 in PCa tissues and cell lines

**Fig. 1:**
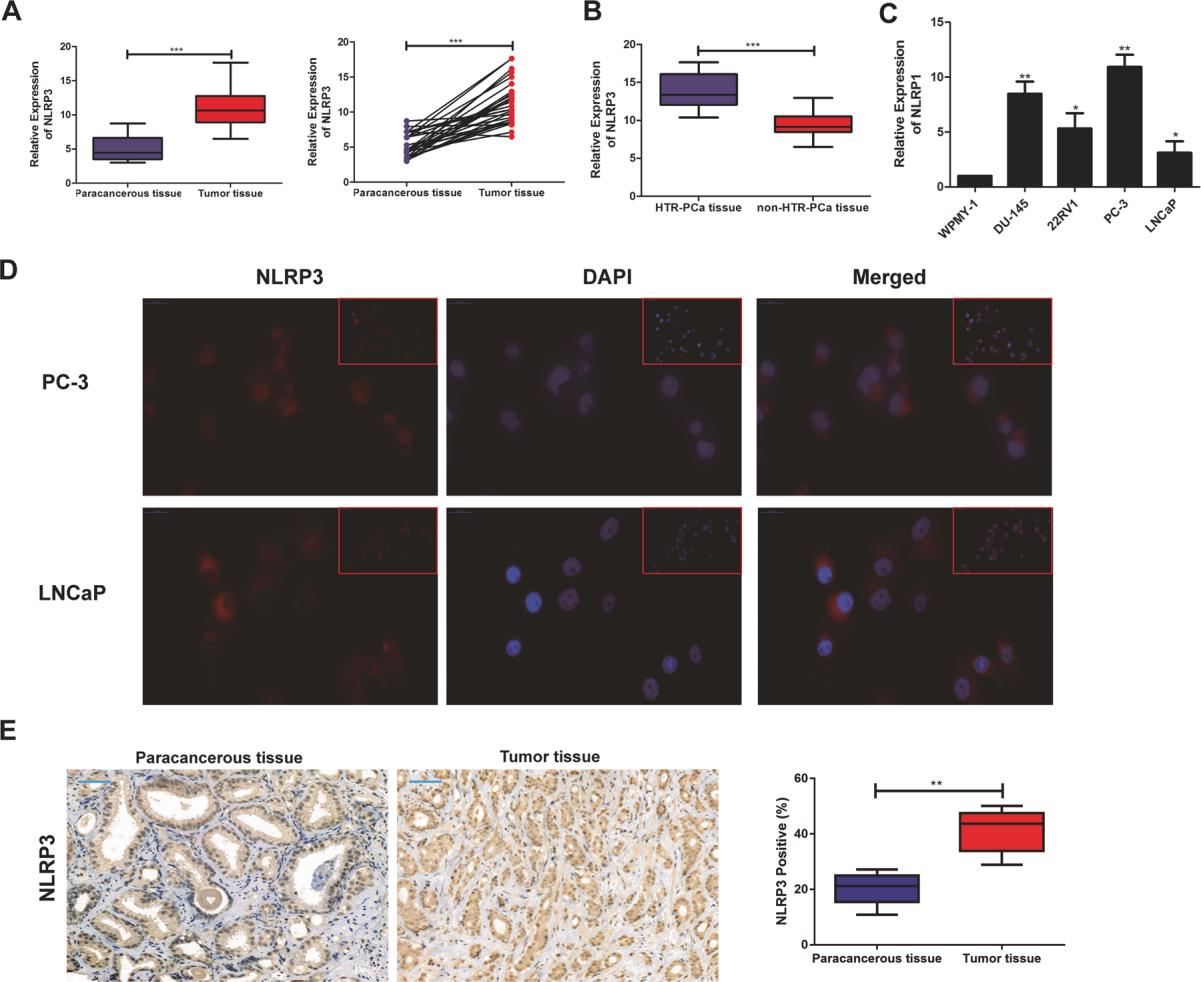
NLRP3 expression level was upregulated in PCa cell lines and tissues. **A** qRT-PCR was used to detect the difference expression of NLRP3 in tumor tissues and corresponding paracancerous ones of PCa patients. **B** qRT-PCR was used to detect the difference expression of NLRP3 in the hormone therapy refractory prostate cancer (HTR-PCa) tissues and the non-hormone therapy refractory prostate cancer (HTR-PCa) ones of these patients. **C** qRT-PCR was used to detect the expression level of NLRP3 in PCa cell lines. **D** The subcellular localization of NLRP3 detected in PC3 and LNCaP cell lines by FISH assay (magnification: ×40). **E** Immunohistochemical staining showed the difference expression of NLRP3 in tumor tissues and corresponding paracancerous ones of PCa patients. Data are mean ± SD, *p < 0.05, **p < 0.01, ***p < 0.001.

The data from PCa patients were complied for investigating the expression level of NLRP3 associated with the malignant progression of PCa. As shown in Fig. 1A, the expression level of NLRP3 significantly upregulated in PCa tissues (*P* < 0.05). Amongst them, the number of patients with the hormone therapy refractory prostate cancer (HTR-PCa) was 12 patients. Therefore, we compared the tissues from HTR-PCa and non-hormone therapy refractory prostate cancer (non-HTR-PCa). The results found that the expression of NLRP3 in the tissues from HTR-PCa was higher than that in the tissues from non-HTR-PCa (Fig. 1B). Then, qRT-PCR also revealed the upregulated expression level of NLRP3 in PCa cell lines. Among them, the relative to high expression in PC3 and low expression in LNCaP were used for subsequent cell function experiments (Fig. 1C). As predicated by the lncATLAS website (http://lncatlas.crg.eu/), NLRP3 was mainly predicted to be localized in the cytoplasm of PCa cell lines, which was further confirmed by Immunofluorescence assay (Fig. 1D). To assess whether NLRP3 was also highly expressed in PCa tissues, a pairs of tumor tissues and matched adjacent non-cancerous tissues of PCa were selected for Immunohistochemical Staining, the results suggested that the expression level of NLRP3 in PCa tissue was significantly upregulated, compared to the paracancerous tissue (Fig. 1E). Taken together, our findings revealed that NLRP3 expression level was significantly high in PCa tissues and cell lines, and it might act as an oncogene in PCa.

### High expression of NLRP3 was correlated with the higher TNM stage and lymph node migration of PCa patients

A total of 30 pairs of PCa tissues and corresponding paracancerous tissues were collected from these PCa patients, and were divided into NLRP3 high-expression group and NLRP3 low-expression group. The associations of NLRP3 expression level with Age, Serum PSA, Gleason score, tumor size, TNM stage, and Lymph node migration of PCa patients were further explored in this study. Table 1 indicated that these patients in NLRP3 high expression showed the higher TNM stage and lymph node migration of PCa patients, in comparison to these patients in NLRP3 low expression. Therefore, these results suggested that NLRP3 might be involved in the malignant progression of PCa.

**Table 1:** Association of NLRP3 expression with clinicopathologic characteristics of prostate cancer.

Variables	Patient number (*N*)	NLRP3 expression		*P*-value
		Low (*n* = 12)	High (*n* = 18)	
Age (years)				0.765
≤60	14	6	8	
>60	16	6	10	
Serum PSA (ng/ml)				0.171
≤10	12	3	9	
>10	18	9	9	
Gleason score				0.879
≤6	12	5	7	
>6	18	7	11	
Tumor size (cm)				0.171
≤2	12	3	9	
>2	18	9	9	
TNM stage				0.044*
I and II	11	7	4	
III and IV	19	5	14	
Lymph node metastasis				0.018*
No	10	7	3	
Yes	20	5	15	

Low/high by the sample median. Pearson *χ*^2^ test.

*P < 0.05 was considered statistically significant.

### LPS + ATP activated NLRP3/caspase-1 inflammasome in PCa cell lines

**Fig. 2:**
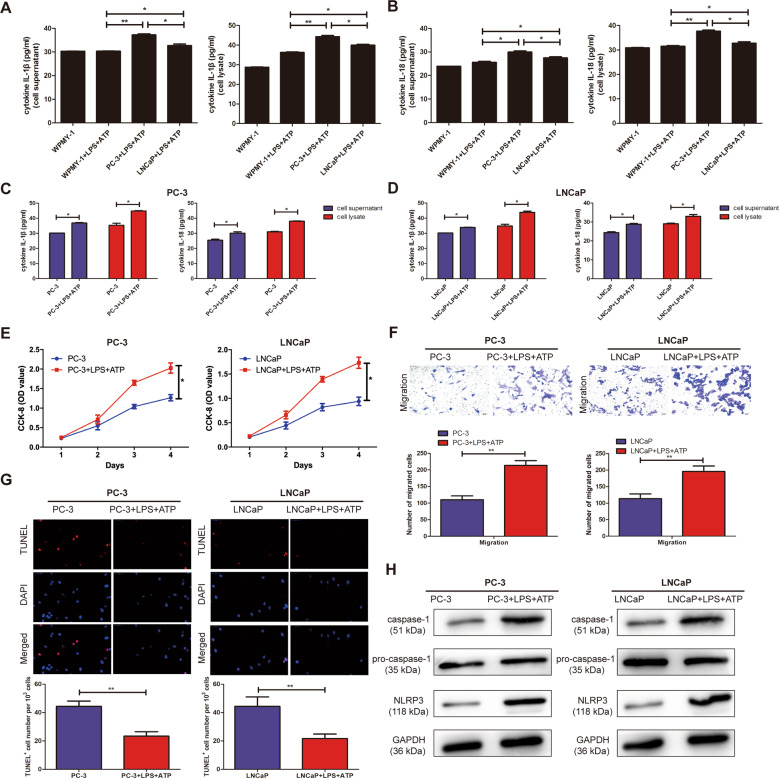
The effects of LPS and ATP on the activation of NLRP3 inflammasome in PC3 and LNCaP cell lines. **A** The concentration of cleaved IL-1β in culture supernatants and lysate were significantly elevated in PC3 cell lines by stimulation of LPS + ATP, not in WPMY-1. **B** The concentration of cleaved IL-18 in culture supernatants and lysate were significantly elevated in LNCaP cell lines by stimulation of LPS + ATP, not in WPMY-1. **C** The expression of IL-1β and IL-18 in the supernatant and lysate of PC3 cell lines were significantly elevated in LPS + ATP group, not in LPS or ATP alone group. **D** The expression of IL-1β and IL-18 in the supernatant and lysate of LNCaP cell lines were significantly elevated in LPS + ATP group, not in LPS or ATP alone group. **E** CCK-8 assay detected the proliferation of PCa cell lines in the stimulation of LPS + ATP. **F** Transwell assay was used to detect the migration ability after the stimulation of LPS + ATP in PC3 and LNCaP cells (magnification: ×40). **G** TUNEL assay was used to detect the apoptosis after the stimulation of LPS + ATP in PC3 and LNCaP cells (magnification: ×40). **H** Western Blot was used to detect the expression level of NLRP3 and caspase-1 after the stimulation of LPS + ATP in PC3 and LNCaP cells. Data are mean ± SD. Scale bar, 20 µm. *p < 0.05, **p < 0.01.

The association between NLRP3 and caspase-1 was determined to investigate whether LPS and ATP trigger the formation of NLRP3 inflammasome in PC3 and LNCaP cells. As shown in Fig. 2A, B, ELISA assay showed that the expression of IL-1β and IL-18 in the supernatant and lysate of PCa cells significantly increased by stimulation of LPS + ATP, compared with WPMY-1. Moreover, the extracellular levels of IL-1β and IL-18 in the supernatant and lysate of PC3 and LNCaP cell lines significantly increased in the LPS + ATP group, compared to the normal cells (Fig. 2C, D). Together, these data demonstrated that LPS + ATP induced the assembly and activation of NLRP3 inflammasome in PC3 and LNCaP cell lines.

### The activation of NLRP3 inflammasome enhanced the malignant progression of PC3 and LNCaP cell lines

As shown in Fig. 2E, CCK-8 assay showed that a significant increase in the proliferation cells was found after the treatment of LPS + ATP, but not LPS or ATP alone. Transwell assay revealed that the migration ability of PCa cell lines was significantly increased in the stimulation of LPS + ATP, compared with not LPS or ATP alone (Fig. 2F). In addition, TUNEL assay was also performed to further investigate the effect of NLRP3 inflammasome on the apoptotic ability. The results indicated that the stimulation of LPS + ATP contributed to significantly decrease the apoptosis in PC3 and LNCaP cell lines (Fig. 2G). The above results showed that NLRP3 inflammasome significantly enhanced the ability of PC3 and LNCaP cells to proliferation and migration; but decreased the apoptotic ability of PCa cell lines. As shown in Fig. 2H, western blotting showed that the expression level of NLRP3 and caspase-1 in the treatment of LPS + ATP were dramatically increased, compared with not LPS or ATP alone (*P* < 0.05).

### Overexpression/knockdown of NLRP3 promoted/inhibited the malignant progression in PCa cell lines

**Fig. 3:**
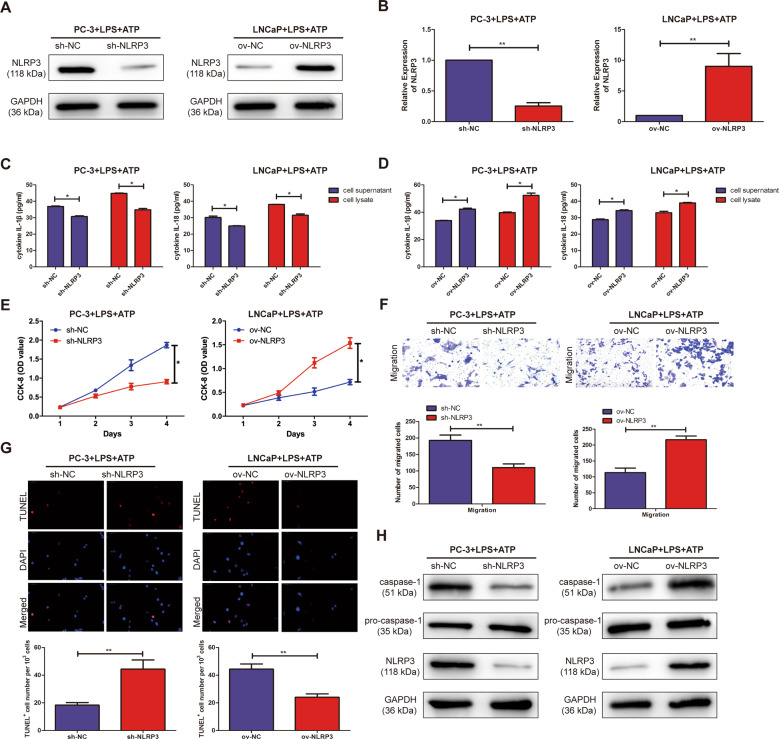
Overexpression/knockdown of NLRP3 promoted/inhibited the malignant progression in PCa cell lines. **A** Western blotting analysis verified the transfection efficiency of NLRP3 after overexpression/knockdown of NLRP3 in PC3 and LNCaP cell lines. **B** qRT-PCR analysis verified the transfection efficiency of NLRP3 after overexpression/knockdown of NLRP3 in PC3 and LNCaP cell lines. **C** The expression of IL-1β and IL-18 in the supernatant and lysate of PC3 and LNCaP cell lines were significantly decreased in sh-NLRP3, not in sh-NC group. **D** The expression of IL-1β and IL-18 in the supernatant and lysate of PC3 and LNCaP cell lines were significantly decreased in ov-NLRP3, not in ov-NC group. **E** CCK-8 assay detected the proliferation of PCa cells in the transfection of NLRP3 overexpression/knockdown vector. **F** Transwell assay was used to detect the migration ability after the transfection of NLRP3 overexpression/knockdown vectors in PC3 and LNCaP cells (magnification: ×40). **G** TUNEL assay was used to detect apoptosis after the transfection of NLRP3 overexpression/knockdown vectors in PC3 and LNCaP cells (magnification: ×40). **H** Western Blot was used to detect the expression level of NLRP3 and caspase-1 after the transfection of NLRP3 overexpression/knockdown vectors in PC3 and LNCaP cells. Data are average ± SD. *p < 0.05, **p < 0.01.

To explore the biological function of NLRP3 inflammasome in PCa cell lines was analyzed by CCK-8, TUNEL assay, and Transwell assay. NLRP3 knockout and overexpression vectors were successfully constructed by western blotting in PC3 and LNCaP cell lines, respectively (Fig. 3A). qRT-PCR assay also confirmed the above results (Fig. 3B). ELISA assay showed that the expression of IL-1β and IL-18 in the supernatant and lysate of PCa cell lines significantly increased/decreased in PC3 and LNCaP cells with overexpression/knockout of NLRP3 by stimulation of LPS + ATP, compared with ov-NC/sh-NC (Fig. 3C, D). It was found by the CCK-8 assay that the proliferation ability of sh-NLRP3 was significantly decreased in PCa cell lines by stimulation of LPS + ATP, compared with sh-NC. However, overexpression of NLRP3 (ov-NLRP3) significantly promoted the proliferation ability of PC3 and LNCaP cell lines, compared with ov-NC (Fig. 3E). Transwell assay revealed that the migration ability of PCa cell lines was significantly decreased or increased in sh-NLRP3 or ov-NLRP3 by stimulation of LPS + ATP, compared with sh-NC or ov-NC (Fig. 3F). TUNEL assay suggested that overexpression/knockdown of NLRP3 was lower/higher apoptosis ability than ov-NC/sh-NC in PC3 and LNCaP cells (Fig. 3G). As shown in Fig. 3H, western blotting showed that compared with ov-NC/sh-NC, the expression level of NLRP3 and caspase-1 in the treatment of LPS + ATP were significantly increased/decreased in ov-NLRP3/sh-NLRP3, respectively. These results suggested that NLRP3/caspase-1 inflammasome could promote the malignant progression in PCa.

### NLRP3 inflammasome exerted oncogenic activity by activating caspase-1 in PCa cells

**Fig. 4:**
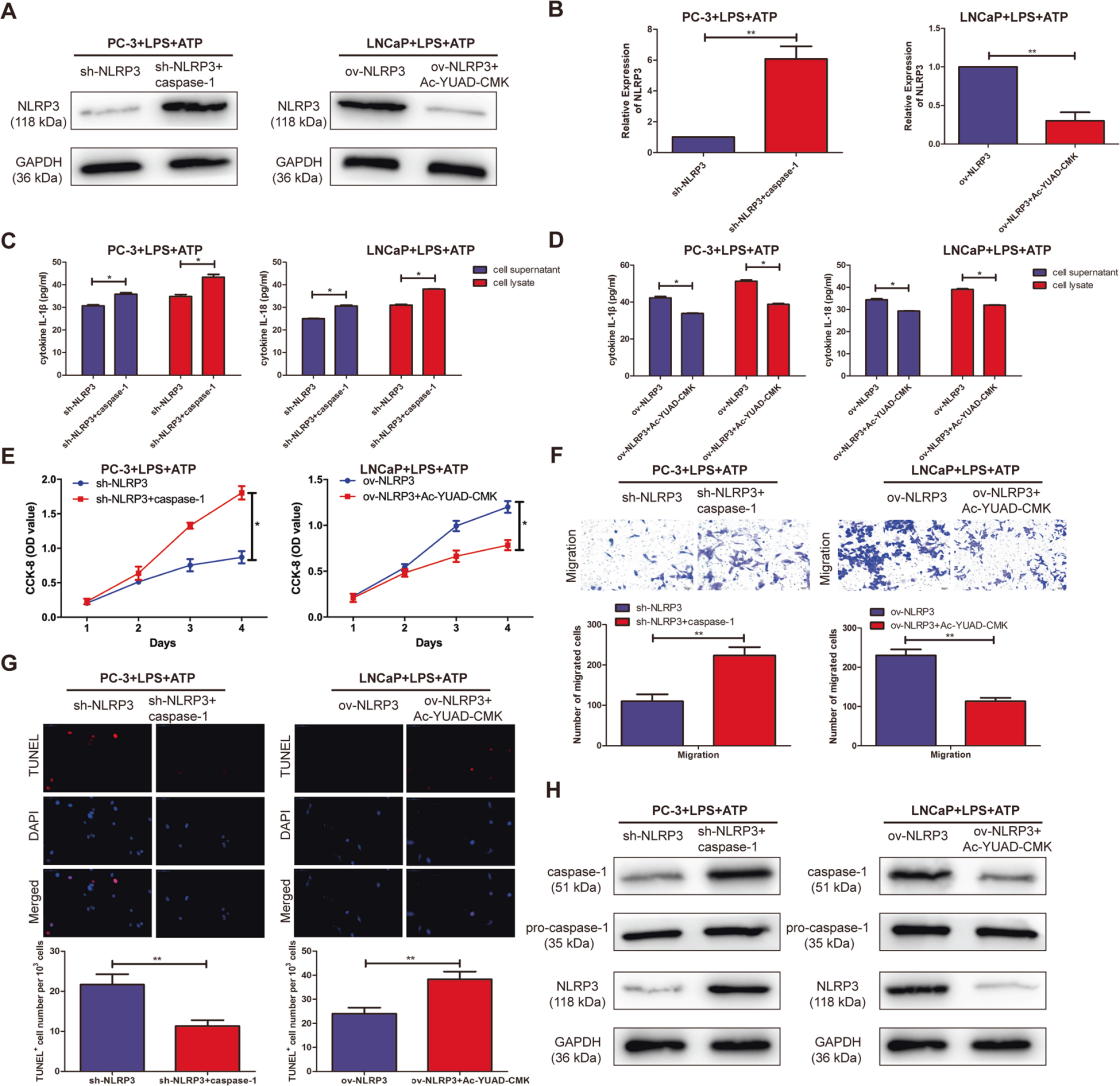
NLRP3 inflammasome exerts oncogenic activity by activating caspase-1 in PCa cell lines. **A** Western blotting analysis verified the transfection efficiency of NLRP3 after sh-NLRP3 + recombinant protein caspase-1 or ov-NLRP3 + Z-YVAD-FMK in PC3 and LNCaP cell lines, respectively. **B** qRT-PCR analysis verified the transfection efficiency of NLRP3 after sh-NLRP3 + recombinant protein caspase-1 or ov-NLRP3 + Z-YVAD-FMK in PC3 and LNCaP cell lines, respectively. **C** The expression of IL-1β and IL-18 in the supernatant and lysate of PC3 and LNCaP cell lines were significantly decreased in sh-NLRP3 + recombinant protein caspase-1, not in sh-NLRP3 alone. **D** The expression of IL-1β and IL-18 in the supernatant and lysate of PC3 and LNCaP cell lines were significantly decreased in ov-NLRP3 + Z-YVAD-FMK, not in ov-NLRP3 alone. **E** CCK-8 assay detected the proliferation of PCa after sh-NLRP3 + recombinant protein caspase-1 or ov-NLRP3 + Z-YVAD-FMK in PC3 and LNCaP cell lines, respectively. **F** Transwell assay was used to detect the migration ability after sh-NLRP3 + recombinant protein caspase-1 or ov-NLRP3 + Z-YVAD-FMK in PC3 and LNCaP cell lines, respectively (magnification: ×40). **G** TUNEL assay was used to detect the apoptosis after sh-NLRP3 + recombinant protein caspase-1 or ov-NLRP3 + Z-YVAD-FMK in PC3 and LNCaP cell lines, respectively (magnification: ×40). **H** Western Blot was used to detect the expression level of NLRP3 and caspase-1 after sh-NLRP3 + recombinant protein caspase-1 or ov-NLRP3 + Z-YVAD-FMK in PC3 and LNCaP cell lines, respectively. Data are average ± SD, *p < 0.05, **p < 0.01.

To further explore the specific regulatory mechanisms in which NLRP3 exactly regulated caspase-1 to promote the malignant progression of PCa. First, to downregulate endogenous caspase-1, PC3, and LNCaP cell lines with caspase-1 inhibitor (Z-YVAD-FMK), western blotting and qRT-PCR analysis demonstrated that NLRP3 expression level was significantly downregulated in PC3 and LNCaP cells treated with Z-YVAD-FMK. In addition, to upregulate endogenous caspase-1, PCa cells with human recombinant protein caspase-1, the results demonstrated that NLRP3 expression was significantly upregulated in PCa cell lines treated with recombinant protein caspase-1 (Fig. 4A, B). ELISA assay showed that the expression of IL-1β and IL-18 in the supernatant and lysate of PCa cells significantly increased in PC3 and LNCaP cells with sh-NLRP3 + recombinant protein caspase-1 by stimulation of LPS + ATP, compared with sh-NLRP3 alone (Fig. 4C). Besides, ov-NLRP3 + Z-YVAD-FMK could significantly reduce the expression of IL-1β and IL-18 in the supernatant and lysate of PCa cells with the stimulation of LPS + ATP, compared with ov-NLRP3 alone (Fig. 4D). Subsequently, the recombinant protein caspase-1 and caspase-1 inhibitor, Z-YVAD-FMK was demonstrated to be able to counteract the proliferation, migration, and apoptosis effects of sh-NLRP3 and ov-NLRP3 in PCa cells by CCK-8, Tranwell assay, and TUNEL assay (Fig. 4E–G). Western blotting showed that compared with sh-NLRP3/ov-NLRP3 alone, the expression level of NLRP3 and caspase-1 in the treatment of LPS + ATP were significantly increased/decreased in sh-NLRP3 + recombinant protein caspase-1/ov-NLRP3 + Z-YVAD-FMK, respectively (Fig. 4H). Therefore, these results suggested that NLRP3 inflammasome could exert an oncogenic activity by activating caspase-1 in PCa cell lines.

### Knockdown of NLRP3 inhibited PCa tumorigenesis in vivo

**Fig. 5:**
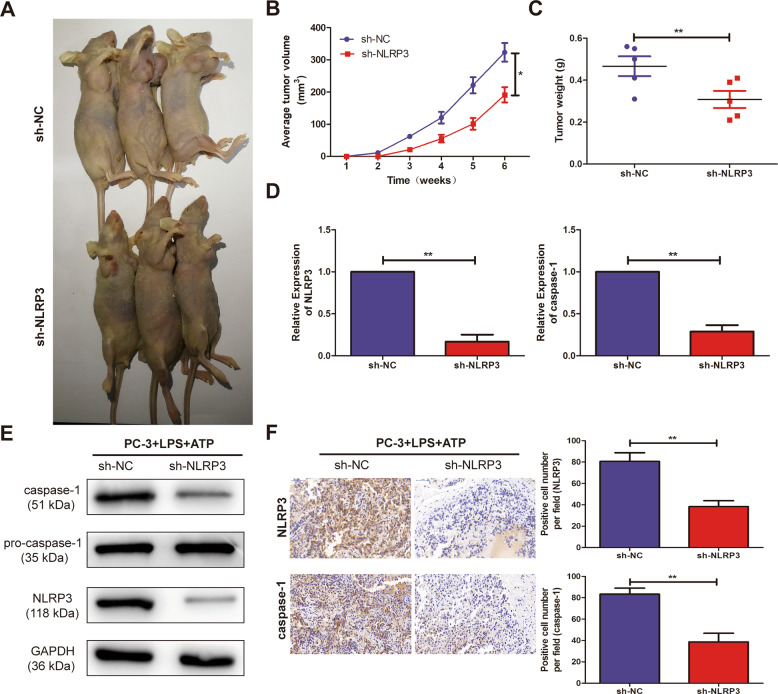
Knockout of NLRP3 inhibited tumorigenic ability in nude mice. **A** Representative pictures of tumor in sh-NC and sh-NLRP3 cell-transplanted mice in PC3 cell line. **B** Tumor volume growth curves were calculated for different nude mice after injection of sh-NC and sh-NLRP3, respectively. **C** Tumor weight growth curves were calculated after injection of sh-NC and sh-NLRP3, respectively. **D** qRT-PCR assay was used to detect the expression level of NLRP3 and caspase-1 in the tumor-forming tissues of nude mice. **E** Western blot was used to detect the expression level of NLRP3 and caspase-1 in the tumor-forming tissues of nude mice. **F** Immunohistochemistry was used to detect the expression level of NLRP3 and caspase-1 in the tumor-forming tissues of nude mice with PC3 cells (magnification: ×40). Data are mean ± SD, *p < 0.05, **p < 0.01.

To further address the biological significance of NLRP3 in tumor growth in vivo, sh-NC or sh-NLRP3 stably transfected PC3 cells were subcutaneously injected into the left flank of nude mice. The results showed that silencing of NLRP3 obviously slowed down the tumor growth of PCa (Fig. 5A, B). Moreover, the weights of nude mice were decreased in sh-NLRP3-transfected PC3 cells, compared with sh-NC (Fig. 5C). In addition, knockdown of NLRP3 triggered a reduction of NLRP3 and caspase-1 expression levels (Fig. 5D) in excised tumor masses. In addition, western blotting showed that compared with sh-NC, the expression level of NLRP3 and caspase-1 were significantly decreased in sh-NLRP3 (Fig. 5E). The immunohistochemistry showed that the NLRP3 and caspase-1 expression levels of sh-NLRP3-transduced PC3 xenografts significantly increased than sh-NC-transduced xenografts (Fig. 5F).

## Discussion

PCa, one of the most common malignant tumors, has the second-highest incidence worldwide and the fifth-highest mortality [1, 3]. Although the incidence of PCa in China is far lower than that in Western countries, its growth is much faster [3]. As China gradually enters the aging society, PCa will become the main tumor “killer” that endangers the health of elderly men [3, 8]. Therefore, the clinical diagnosis and treatment of PCa and its basic study have entered an urgent stage [4, 6]. Previous studies have shown that inflammasomes induced by microbial or viral infections, which sense exogenous and endogenous dangers and initiate inflammatory responses, play a vital role in tumorigenesis by the production of cytokines, chemokines, and extracellular matrix [12, 14, 15]. However, new evidence has been found that it also leads to most stages of cancer development and interferes with immune system suppression of cancer cells and weak responses to treatment [9, 10, 13].

In inflammasome, NLRP3 is widely expressed in epithelial cells, macrophages, keratinocytes, and dendritic cells [16, 17]. NLRP3 inflammasome is characterized by extensive tissue expression profile, cell specificity, and strong tissue, and has a good prospect in molecular diagnosis and targeted therapy of tumors [18, 20]. Based on the PCa tissue sequencing of the next generation of Chinese population, further explore the PCa tumor-associated inflammatory bodies, study their molecular characteristics and functional mechanisms, and discover the NLRP3 molecule for the early diagnosis of PCa, which is helpful to better understand the biological behavior of PCa cells and find new targets for the treatment of prostate cancer. It provides the basis for clinical diagnosis and new drug development of prostate cancer [21]. The activation of NLRP3 inflammasome commonly includes priming with a TLR agonist (such as LPS) and activating with a second stimulus (such as ATP) [22]. The results showed that the activation of NLRP3 by the effects of LPS + ATP increased the proliferation and migration activity, but decreased the apoptosis level in PC3 and LNCaP cells.

NLRP3 has been known for a long time and can be involved in many physiological and pathological processes, including tumor progression [19, 20, 23]. In our study, to explore the role of NLRP3 inflammasome in the malignant progression of PCa, qRT-PCR assay was performed and detected the upregulated the expression level of NLRP3 in PCa tissues and cell lines. Meanwhile, we found that NLRP3 could be used as an indicator of the malignant progression of PCa, and thus serve as an oncogene in PCa. The results by CCK-8, TUNEL and Transwell assay showed NLRP3 inflammasome significantly enhanced the ability of PC3 and LNCaP cells to proliferation and migration; but decreased the apoptotic ability of PCa cell lines. The results of the tumor formation in nude mice showed that silencing of NLRP3 significantly slowed down the tumor growth of PCa. However, its specific molecular mechanism still remains unclear.

As far as we know, one of the mechanisms of NLRP3 inflammasome is the recruitment of protein or RNA to target genes, thereby exerting its biological significance indirectly [23, 24]. To elucidate the possible mechanisms by which NLRP3 inflammosomes are involved in the pathogenesis of PCa, we performed a subcellular fractionation assay. The results showed that NLRP3 was mainly located in the nucleus of PC3 and LNCaP cells, reflecting its regulation at the transcriptional level. qRT-PCR and Western blot analysis showed that the level of caspase-1 protein in PC3 and LNCaP cells decreased/increased after NLRP3 knockdown/overexpression. Based on the fact that then NLRP3 inflammasome activates caspase-1 to release these cytokines from cells, we became interested in whether caspase-1 deficiency would result in poor mobilization [25]. To conclude, NLRP3 epigenetically increased the expression level of caspase-1 in PCa cell lines via the activation of caspase-1. In this study, the upregulated expression level of NLRP3 in PCa tissues and cell lines was detected, which was positively correlated with that of caspase-1. Furthermore, to investigate the impact of the interaction between NLRP3/caspase-1 inflammasome in the development of PCa, the reverse experiments were performed in vitro and in vivo. It was found that NLRP3 inflammasome could serve as the tumor-promoting effect by the activation of caspase-1 in PCa. In summary, our research defined a molecular mechanism for promoting the malignant progression of PCa by NLRP3 inflammasome through the activation of caspase-1. The identification and validation of our study provided a novel insight into the research and clinical management of PCa. Therefore, this newly identified NLRP3 inflammasome might serve as a prognostic biomarker and a potential therapeutic target for PCa.

## Methods

### Patients and PCa samples

Tumor tissues and paracancerous ones of 30 PCa patients undergoing radical prostatectomy were collected in this study. All subjects had not received any preoperative radiotherapy or chemotherapy. The pathological classification and TNM staging criteria of PCa are implemented in accordance with the staging criteria of International Union Against Cancer (UICC). Each pair of PCa specimens was from the same patient after pathological examination. Then, the tissue fragments were immediately transferred into liquid nitrogen and stored at −80 °C before use. All PCa patients in this study had been fully signed the informed consent. In addition, this study has been approved by the Ethics Committee of Nanjing Medical University.

### Reagents and cell lines

Four human-derived PCa cell lines (PC3, DU-145, 22RV1, LNCaP) and the human normal prostate stromal immortalized cell (WPMY-1) provided by ATCC were cultured in F-12k medium and 1640 medium (Life Technologies, USA) containing 10% fetal bovine serum (10% FBS), 100 U/ml penicillin, and 100 mg/ml streptomycin. These cells were cultured in a humidified air atmosphere at 37 °C with 5% CO_2_.

Lipopolysaccharide (LPS) and adenosine triphosphate (ATP) were purchased from Sigma-Aldrich (St. Louis, MO, USA). For upregulation of caspase-1, human recombinant protein caspase-1 was purchased from BioVision (San Francisco, USA) following the manufacturer’s instructions. The caspase-1 inhibitor benzyloxycarbonyl-tyrosine-valine-alanine-aspartate-fluoromethyl ketone (Z-YVAD-FMK) was purchased from PromoCell (Heidelberg, Germany). To inhibit the activation of caspase-1, these cells were pre-treated with 10 µmol/l Z-YVAD-FMK for half an hour prior to LPS stimulation, respectively. These Cells were stimulated by 1 µg/ml LPS for 8 h with or without 5 mmol/l ATP for the last half an hour.

### Enzyme-linked immunosorbent assay (ELISA)

LNCaP and PC3 cells were seeded in 24-well plates overnight. For NLRP3 inflammasome activation, these cells were stimulated by 1 µg/ml LPS for 8 h with or without 5 mmol/l ATP for the last half an hour. After that, cell supernatants and lysates were collected, and centrifuged at 300 × *g* for 8 min at 4 °C for elimination of cells detached due to cell death, to generate cell-free medium preparations. Then, the supernatants and lysates were concentrated by centrifugation at 12,000 × *g* for 30 min at 4 °C through a column with a cut-off of 10 kDa (Microcon; Merck-Millipore, Darmstadt, Germany). The levels of IL-1β and IL-18 in cell culture supernatants and lysates were measured by human ELISA kits from R&D Systems (Minneapolis, MN, USA) and MBL (Nagoya, Japan), according to the manufacturer’s instructions.

### Transfection

Lentivirus packaging cells were transfected with LV3-pGLV-h1-GFP-puro vector (GenePharma, China) containing either NLRP3 knockdown (sh-NLRP3) or NLRP3 overexpression (ov-NLRP3) and a negative control sequence (sh-NC and ov-NC), respectively. Lentiviral transduction was performed in the PCa cell lines (PC3 and LNCaP). The pools of stable transductants were generated by selection using puromycin (4 μg/ml) for 2 weeks, and these cells were collected for cell function experiments.

### Immunohistochemical staining

The tissue sections were dewaxed, rehydrated, and washed 3 times for 5 min phosphate-buffered saline (PBS). After slides were microwaved for 20 min and allowed to cool for 1 h at room temperature, endogenous peroxidase activity was blocked in all sections by incubating the sections in 3% H_2_O_2_ for 15 min. The sections were incubated with Anti-NLRP3 and Anti-caspase-1 monoclonal antibodies (Cell Signaling Technology, USA) overnight at 4 °C. Next day, the slides were washed and incubated with a horseradish peroxidase (HRP)-conjugated anti-goat secondary antibody (Cell Signaling Technology, USA) at a 1:100 dilution for 1 h. After stained by DAB, the sections were observed under light microscopy.

### Immunofluorescence assay

The human PCa cell lines (PC3 and LNCaP) were fixed with 4% paraformaldehyde for 30 min, permeabilized with 0.5% Triton X-100 for 15 min, and incubated with 5% bovine serum albumin (BSA) in PBS for 1 h at room temperature. Subsequently, cells were incubated with primary antibodies against NLRP3 (1:200, Abcam, London, UK) overnight at 4 °C. After three washes with PBS (5 min per wash), cells were incubated with anti-rabbit (1:500, Invitrogen) for 1 h at room temperature. After three washes with PBS, nuclei were stained with Hoechst 33342 (Beyotime, Nantong, China) for 30 min. Immunofluorescence images were captured using a Zeiss LSM5 Live confocal laser scanning microscope (Carl Zeiss, Jena, Germany).

### CCK-8 assay

A Cell Counting Kit-8 assay (Dojindo Laboratories, Japan) was used to estimate the proliferation potential. The cells were inoculated in 96-well plates with 3000 cells per well. Add 200 mL complete medium to each well (5 Wells parallel to each group). The cells were cultured in 37 °C, 5% CO_2_ incubator. 10 mL CCK-8 solution (5 mg/mL) was added to each well at 0, 1, 2, 3, and 4 d, respectively, and cultured continuously for 4 h. The absorbance of each well was measured at 450 nm using a micro-plate reader, and the cell growth curve was plotted.

### TdT-mediated dUTP nick-end labeling (TUNEL) assay

TUNEL assays were performed on 4% paraformaldehyde-fixed sections by using In Situ Apoptosis Detection Kit (Roche, Basel, Switzerland) in accordance with the manufacturer’s instructions. The nucleus of any apoptotic cells would exhibit brown stains under a fluorescence microscope and was quantitatively counted manually in a blinded fashion. The results were presented as TUNEL-positive cells per 10^3^ germ cells. The number of TUNEL-positive versus total cell nuclei was counted in five randomly chosen high-power fields (×400) containing 10^3^ cells on each slide to calculate an apoptotic index.

### Transwell migration assay

Transwell chamber inserts with un-coated Matrigel (migration) were used for transwell migration assay. The transfected cells (PC3 and LNCaP) were seeded into the upper of the 24-well cell culture inserts coating with 200 μl serum-free medium. In addition, 400 μl complete medium containing 10% fetal bovine serum was added to the base of the insert to induce cell migration for 24 h. After incubation, these cells in the bottom of the chamber were removed with cotton swabs, whereas these cells on lower filter surfaces were fixed with 4% formaldehyde and stained with crystal violet. The number of migrated cells was counted under a microscope, according to the manufacturer’s instructions.

### Quantitative real-time PCR (qRT-PCR)

The total RNA in both PCa cell lines (in vitro) and PCa samples (in vivo) was extracted using an RNeasy Mini Kit (ThermoFisher Scientific, USA) according to the manufacturer’s protocol. Each sample was lysed with 1 ml TRIzol. The initially extracted RNA was treated with DNase I to remove genomic DNA and repurify the RNA. With the help of the Prime Scirpt Reverse Transcription Kit (Takara) instructions, RNA reverse transcription was performed, real-time PCR was performed according to the SYBR® Premix Ex TaqTM (Takara) Kit instructions, and the PCR reaction was performed using the StepOne Plus Real-time PCR System (Applied Biosystems, Foster City, CA, USA) system. Each sample was repeated with 3 replicate wells repeated twice. Bio-Rad PCR instrument and software iQ5 2.0 were used to analyze and process the data. GAPDH was used as internal parameters, and NLRP3 and caspase-1 expression was calculated by 2−ΔΔCt method. The following primers were used for qRT-PCR reactions:

NLRP3:

forward, 5′-ATGTGGGGGAGAATGCCTTG-3′,

reverse, 5′-TTGTCTCCGAGAGTGTTGCC-3′;

caspase-1:

forward, 5′-GCAATGAAGACGAAGGCGAC-3′,

reverse, 5′-GTGCCCGTGCGAGATTTTAG-3′;

GAPDH:

forward, 5′-GAAGGTGAAGGTCGGAGTC-3′,

reverse, 5′-GAAGATGGTGATGGGATTTC-3′.

### Western blot

The transfected cells or tissues were lysed using cell lysis buffer, shaken on ice for 30 min, and centrifuged at 14,000 × *g* for 15 min at 4 °C. After thermal denaturation, the proteins were stored in the refrigerator −20 °C. Total protein concentration was calculated by the BCA Protein Assay Kit (Pierce, Rockford, USA). Anti-NLRP3, anti-caspase-1, and anti-pro-caspase-1 monoclonal antibodies were purchased from Santa Cruz, USA, while the horseradish peroxidase-labeled goat anti-rabbit secondary antibody was purchased from Genscript. GAPDH was used as internal reference control. Protein samples were separated using SDS-PAGE electrophoresis and then transferred to a PVDF membrane. The membrane was sealed with 5% skimmed milk for 1 h, add primary antibody at 4 °C overnight, and re-warmed for 2 h the next morning. The membranes were washed with Tris-buffered saline with Tween 20 and incubated with secondary antibody for 1 h. The image was semi-quantitatively analyzed using alpha SP image analysis software.

### In vivo xenograft model

The Animal Ethics and Use Committee of Nanjing Medical University approved the tumor-forming experiment in nude mice. 8-week-old male nude mice were purchased from the animal center and randomly divided into two groups (5 in each group). PC3 cells with sh-NLRP3 and sh-NC were injected subcutaneously into the axilla of mice. Tumor size was monitored every 1 week; then, after 6 weeks, the mice were sacrificed. The tumor volumes were calculated using the following formula: tumor volume = (width^2^ × length)/2.

### Statistical analysis

Statistical analysis was performed using SPSS 22.0 software. Univariate analysis was performed using the *χ*^2^ test and the exact probability Fisher test. Multivariate analysis was performed using COX regression analysis, while the survival of these PCa patients was analyzed using the Kaplan–Meier method. Differences between groups were analyzed by the *t*-test. Pearson correlation test was applied for evaluating the associations between the expression levels of NLRP3 and caspase-1 in PCa specimens. These data were expressed as mean ± standard deviation, and *P* < 0.05 was considered to be statistically significant.
